# Monocyte-driven IFN and TNF programs orchestrate inflammatory networks in antisynthetase syndrome-associated interstitial lung disease

**DOI:** 10.3389/fimmu.2025.1652999

**Published:** 2025-10-23

**Authors:** Yu Fan, Weijin Zhang, Miaotong Su, Shaoyu Zheng, Jianqun Lin, Kedi Zheng, Fengcai Shen, Guohong Zhang, Yukai Wang

**Affiliations:** ^1^ Department of Pathology, Shantou University Medical College, Shantou, China; ^2^ Department of Rheumatology and Immunology, Shantou Central Hospital, Shantou, China; ^3^ Department of Biomedical Sciences, University of Sassari, Sassari, Italy

**Keywords:** antisynthetase syndrome, interstitial lung disease, monocytes, interferon-gamma, TNF signaling

## Abstract

**Objective:**

Antisynthetase syndrome-associated interstitial lung disease (ASS-ILD) exhibits clinical heterogeneity and progression, with unclear immunopathogenic mechanisms. This study aimed to define the cell type-specific interferon immune signatures and transcriptional networks underlying ASS-ILD.

**Methods:**

Single-cell RNA sequencing (scRNA-seq) was performed on peripheral blood mononuclear cells (PBMCs) from three treatment-naive ASS-ILD patients and three healthy controls (67,421 cells). A comprehensive analysis was conducted in conjunction with an external cohort, encompassing 126,026 cells. The analytical pipelines included the following: AUCell for interferon-stimulated gene (ISG) activity scoring, Seurat for clustering, Monocle for trajectory inference, and CellChat for cell–cell communication. The inference of transcription factor activity was facilitated using decoupleR software.

**Results:**

Monocyte-specific ISG activity was identified and validated in an integrated cohort of 126,026 cells. Among the six monocyte subsets, mono2 exhibited elevated *IFNG* expressions and a preferential inflammatory trajectory, marked by upregulated innate and adaptive immune pathways. Cell-cell interaction modeling revealed dysregulated type II interferon (IFN-II) and tumor necrosis factor (TNF) signaling, with mono2, NK, and CD8^+^ T cells as key signal transmitters. Regulatory network analysis revealed that the transcription factors *ETV5*, *IRF5*, *IRF7*, *RORB*, *RORC*, and *SMAD1* drive inflammatory and profibrotic signatures via the IL-17, JAK-STAT, and TGF-β pathways.

**Conclusions:**

This study identifies monocytes as central orchestrators of immune dysregulation in ASS-ILD, highlighting IFN/TNF signaling and associated transcriptional regulators as therapeutic targets.

## 1 Introduction

Antisynthetase syndrome (ASS) is a clinically heterogeneous subset of idiopathic inflammatory myopathies (IIMs), defined by the presence of myositis-specific autoantibodies (MSAs) targeting aminoacyl-tRNA synthetases, including Jo-1, EJ, OJ, PL-7, PL-12, KS, Hs, and Zo (1). ASS typically presents with a triad of inflammatory myopathy, arthritis, and interstitial lung disease (ILD), though phenotypic variability is considerable (2). Among these manifestations, pulmonary involvement is both the most frequent and prognostically significant, with ILD occurring in up to 85% of patients. Histopathological patterns are commonly nonspecific interstitial pneumonia (NSIP) or organizing pneumonia (OP) (3). ILD may precede, coincide with, or follow muscular and articular symptoms, and in many patients, represents the initial and dominant clinical feature (4).

Despite the high prevalence and clinical burden of ILD in ASS, the underlying immunopathogenic mechanisms remain poorly understood, limiting the development of targeted therapies. Notably, growing evidence implicates interferon (IFN) signaling in the pathogenesis of IIMs, with disease subtype-specific IFN signatures emerging as a central theme. For instance, anti-MDA5^+^ dermatomyositis is characterized by type I IFN activation, while ASS and inclusion body myositis more prominently feature type II IFN responses (5).

The induction of IFNs and subsequent expression of interferon-stimulated genes (ISGs) are orchestrated by multiple immune and non-immune cell types, including monocytes, neutrophils, plasmacytoid dendritic cells, macrophages, and epithelial cells (6). Dysregulated ISG expression has been associated with disease severity in various autoimmune contexts (7). However, the cell type-specific distribution and regulatory dynamics of IFN-related programs in ASS-ILD—particularly at single-cell resolution—remain largely undefined.

In this study, we applied single-cell RNA sequencing (scRNA-seq) to profile peripheral blood mononuclear cells (PBMCs) from treatment-naive patients with ASS-ILD. Through integrative computational analyses, we aimed to identify pathogenic IFN-related signatures and delineate the cellular and transcriptional networks driving immune dysregulation in ASS-ILD. By resolving immune heterogeneity and uncovering mechanistic insights into monocyte-mediated inflammatory programs, our study provides a framework for understanding disease pathogenesis and informing future therapeutic strategies.

## 2 Materials and methods

### 2.1 Patient selection

Single-cell RNA sequencing analysis was conducted on PBMC samples from three ASS-ILD treatment-naive patients and three healthy individuals recruited at Shantou Central Hospital. Healthy individuals were selected to be sex-, ethnicity-, and age-matched. Informed consent was obtained from all the subjects. The diagnosis of ASS-ILD was made by a multidisciplinary team comprising an expert rheumatologist and two experienced radiologists specializing in chest CT. ASS was diagnosed in accordance with the criteria proposed by Solomon et al. (8). Patients with other identifiable causes of ILD, including those with medication-related lung injury, malignancy, or environmental and occupational exposures, were excluded from the study. **Supplementary Table 1** provides a detailed description of the clinical presentation and laboratory characteristics of the patients, while **Supplementary Figure 1** offers a visual representation of the CT images of the patients. All samples were collected in accordance with the ethical requirements and regulations of the Ethics Committee of Shantou Central Hospital. Informed consent was obtained from all the subjects, and the studies were conducted under approval (approval number:〔2022〕KY-006).

Furthermore, a single-cell RNA sequencing dataset [GSE190510 (9)] from the Gene Expression Omnibus (GEO, http://www.ncbi.nlm.nih.gov/geo/) was incorporated, comprising eight PBMC samples from five ASS-ILD patients and three healthy individuals.

### 2.2 Single-cell RNA sequencing

Single-cell sequencing was conducted via the 10x Genomics platform (1). Preparation of single-cell suspensions: The isolation of peripheral blood mononuclear cells (PBMCs) from undiluted human blood was conducted via Histopaque solution (Sigma–Aldrich, St. Louis, MO). A total of 10 µl of the suspension was counted under an inverted microscope with a hemocytometer. The number of live cells was determined via the Trypan blue method (2). Construction of single-cell libraries: Chromium Single-cell 3’ Reagent v3 kits were used to prepare barcoded single-cell RNA sequencing libraries in accordance with the manufacturer’s instructions. The isolated PBMCs were encapsulated via microfluidics technology and barcoded with a unique molecular identifier. cDNA was prepared in accordance with the manufacturer’s specifications (3). Single-cell RNA sequencing library preparation and sequencing: cDNA libraries were sequenced on an Illumina HiSeq PE150 system (4). Raw data processing and quality control: The data were demultiplexed via Cell Ranger software (version 3.1.0), which generated FASTQ files, which were aligned to a human reference genome (GrCh38). The Cell Ranger software generated a unique raw molecular identifier count matrix, which was subsequently converted into a Seurat object via the R package Seurat (version 5.0.1). Cells with doublets and low quality were removed based on the number of unique molecular identifiers (UMIs) and the proportion of mitochondrial gene expression. The genes were filtered on the basis of the number of cells in which they were expressed, and the cells were filtered based on the number of genes expressed in them. The data were subsequently normalized by log normalization, and the top 2000 highly variable genes were selected based on mean expression and variance. All genes were scaled via the ScaleData function, and principal component analysis (PCA) downscaling was performed. The cells were subsequently clustered via the FindNeighbors and FindClusters functions to obtain cell subgroups, and the cells were subsequently annotated. Batch correction was performed via the Harmony algorithm.

### 2.3 Identification of functional cellular subsets within the major cell clusters

The identification of differentially expressed genes and specific marker genes for each cellular subset was achieved by employing Seurat’s FindAllMarkers() function with the parameter “test.use = wilcox” by default under the RNA assay. The definition of each cell subcluster was based on the expression of canonical markers.

### 2.4 Differential expression analysis and functional enrichment analysis

To identify the genes that were upregulated in different cell types or different disease states, the FindMarker function (Logfc.threshold = 0.25, Wilcoxon test) in Seurat was employed. To investigate the biological functions and pathways associated with the differentially expressed genes (DEGs, log2-fold change (FC) > 0.25, adjusted *p* value < 0.05), Gene Ontology Biological Process (GOBP) (10) functional enrichment and Kyoto Encyclopedia of Genes and Genomes (KEGG) (11) pathway analyses were conducted via the clusterProfiler package (version 4.8.3) (12).

### 2.5 Using AUCell to calculate scores of interferon-related genes

The DEGs of each cluster were then used as input to generate ISGs via the Interferome database (https://interferome.org/interferome/home.jspx). This process yielded 235 ISG gene sets (**Supplementary Table 2**), which were subsequently used for ISG scoring via the AUCell R package (version 1.24.0) (13). The ISG set was then employed as the input data for the calculation of the area under the curve (AUC) value. The AUC values were then utilized to construct gene expression rankings for each cell. The AUC provides an estimate of the proportion of genes within the gene set that are highly expressed in each cell. The number of expressed genes in a cell was positively correlated with the AUC value. Consequently, cells that express a greater number of genes from the gene set will have higher AUC values than cells that express fewer genes. The function “AUCell_exploreThresholds” was employed to ascertain the threshold that could be utilized to consider the present gene set active. The cell clustering UMAP embedding was subsequently colored according to the AUC score of each cell, thereby indicating which cell clusters were active in the ISG gene set.

### 2.6 Using gene set variation analysis to identify the functions of cell subsets

Human gene sets from GOBP were retrieved via the msigdbr package (version 7.5.1). We subsequently applied GSVA (14) with the GSVA package (version 1.50.0) to assign pathway activity estimates to individual cells.

### 2.7 Cell–cell interaction analysis

The cell–cell interactions between different cell types were evaluated via CellChat (version 1.6.0, R package). CellChat utilizes gene expression data as the fundamental input to model the probability of cell-to-cell communication by integrating gene expression data with an existing database comprising known interactions between signaling ligands, receptors, and their cofactors (15). Normalized count data from each condition were used to create a CellChat object, and the recommended preprocessing functions for the analysis of individual datasets were applied with default parameters.

### 2.8 Gene set enrichment analysis

Gene set enrichment analysis (GSEA) of monocytes from ASS-ILD patients and HCs was performed via the clusterProfiler (version 4.12.6) R software package to analyze the potential biological pathways of monocytes in ASS-ILD. Permutations were set to 10,000 to obtain normalized enrichment scores (NESs) in GSEA. Gene sets with an adjusted P value <0.05 were considered to be significantly enriched. The enrichplot (version 1.24.2) and ggplot2 (version 3.5.1) R packages were employed to display the enrichment results.

### 2.9 Transcription factor activity inference from scRNA-seq

Transcription factor (TF) activity was inferred for monocytes via a univariate linear model (ULM) in the R package decoupleR (version 2.12.0) (16), with CollecTRI (Collection of Transcriptional Regulatory Interactions) serving as the reference. CollecTRI is a comprehensive resource that contains a curated collection of TFs and their transcriptional targets, compiled from 12 different resources (17).

### 2.10 Gene expression validation by qRT-PCR and external dataset analysis

We additionally collected PBMC samples from 5 ASS-ILD patients and 5 matched healthy controls. RNA was extracted using the Trizol method, and cDNA was synthesized using Takara’s reverse transcription kit (cat#RR092A) from Takara Bio Inc. (Shiga, Japan). Semi-quantitative PCR was performed using MCE’s SYBR Green qPCR Master Mix (cat# HY-K0501) from MCE (Shanghai, China), and the results were analyzed using the 2−ΔΔCt method with β-actin as the reference gene for normalization. The primers for each gene are as follows:

β-actin: Forward: GGGAAATCGTGCGTGACATT, Reverse: GGAAGGAAGGCTGGAAGAGT.
IFNG: Forward: TCGGTAACTGACTTGAATGTCCA, Reverse: TCGCTTCCCTGTTTTAGCTGC.
TNF: Forward: GAGGCCAAGCCCTGGTATG, Reverse: CGGGCCGATTGATCTCAGC.
IRF7: Forward: GCTGGACGTGACCATCATGTA, Reverse: GGGCCGTATAGGAACGTGC.
NFKBIZ: Forward: GATTCGTTGTCTGATGGACCTG, Reverse: CGTTGGTGTTTGAGGTGGT.

Additionally, we incorporated an external bulk RNA-seq dataset (GSE220915 (18)), which includes muscle biopsy samples from 18 ASS patients and 33 healthy controls, to validate the expression of key genes.

### 2.11 Statistical analyses

All statistical analyses were performed using R software (version 4.3.2) or GraphPad Prism (version 10.4.0). Normally distributed data are expressed as the mean ± standard deviation and compared using unpaired t-tests. Non-normally distributed data are presented as median (interquartile range) and analyzed using the Mann-Whitney U test. A *p* value of less than 0.05 was considered statistically significant.

## 3 Results

### 3.1 Monocyte subsets in ASS-ILD exhibit enhanced interferon activation

**Figure 1:**
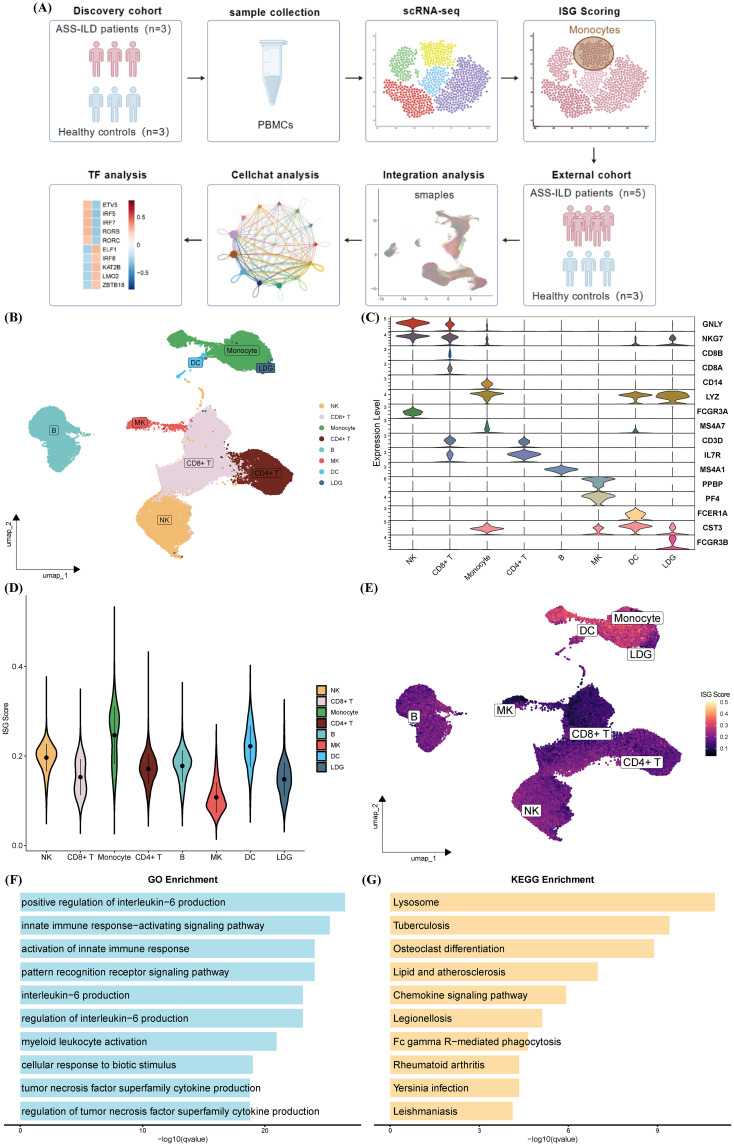
Characterization of PBMC subclusters and interferon activity in ASS-ILD patients compared with healthy controls in the discovery cohort. **(A)** Schematic of the study design. Created with BioGDP.com (38). **(B)** UMAP plot showing eight distinct immune cell populations in PBMCs from three ASS-ILD patients and three healthy controls (HCs). **(C)** Violin plots of canonical marker gene expression used for cell type annotation. **(D, E)** AUCell-based ISG score analysis shown by violin plots **(D)** and UMAP projection **(E)**, highlighting monocytes as the immune subset with the highest ISG activity. **(F, G)** Functional enrichment of monocyte-specific genes, illustrating the top 10 enriched Gene Ontology Biological Process (GOBP) terms **(F)** and KEGG pathways **(G)**.

To elucidate the immunopathological landscape of ASS-ILD, we first analyzed a discovery scRNA-seq cohort comprising peripheral blood mononuclear cells (PBMCs) from three treatment-naive ASS-ILD patients and three healthy controls (HCs), yielding a total of 67,421 cells. Unsupervised clustering followed by UMAP visualization (**Figures 1A, B**) identified eight major immune cell types: CD4^+^ T cells, CD8^+^ T cells, monocytes, natural killer (NK) cells, B cells, low-density granulocytes (LDGs), megakaryocytes (MKs), and dendritic cells (DCs), confirmed by canonical marker gene expression (**Figure 1C**).

To assess interferon activity, AUCell scoring based on 235 curated interferon-stimulated genes (ISGs) from the Interferome database revealed significantly elevated ISG scores in monocytes compared with other immune subsets (**Figures 1D, E**). Gene ontology (GO) and KEGG pathway enrichment further demonstrated that monocytes were enriched for pathways associated with innate immune activation (e.g., IL-6 and TNF production, myeloid cell activation), lysosomal processing, mycobacterial infection, and autoimmune inflammation (**Figures 1F, G**). These findings indicate that monocytes are a key site of interferon-driven immune activation in ASS-ILD.

### 3.2 Integrated cross-cohort analysis uncovers functional heterogeneity among monocyte subsets

**Figure 2:**
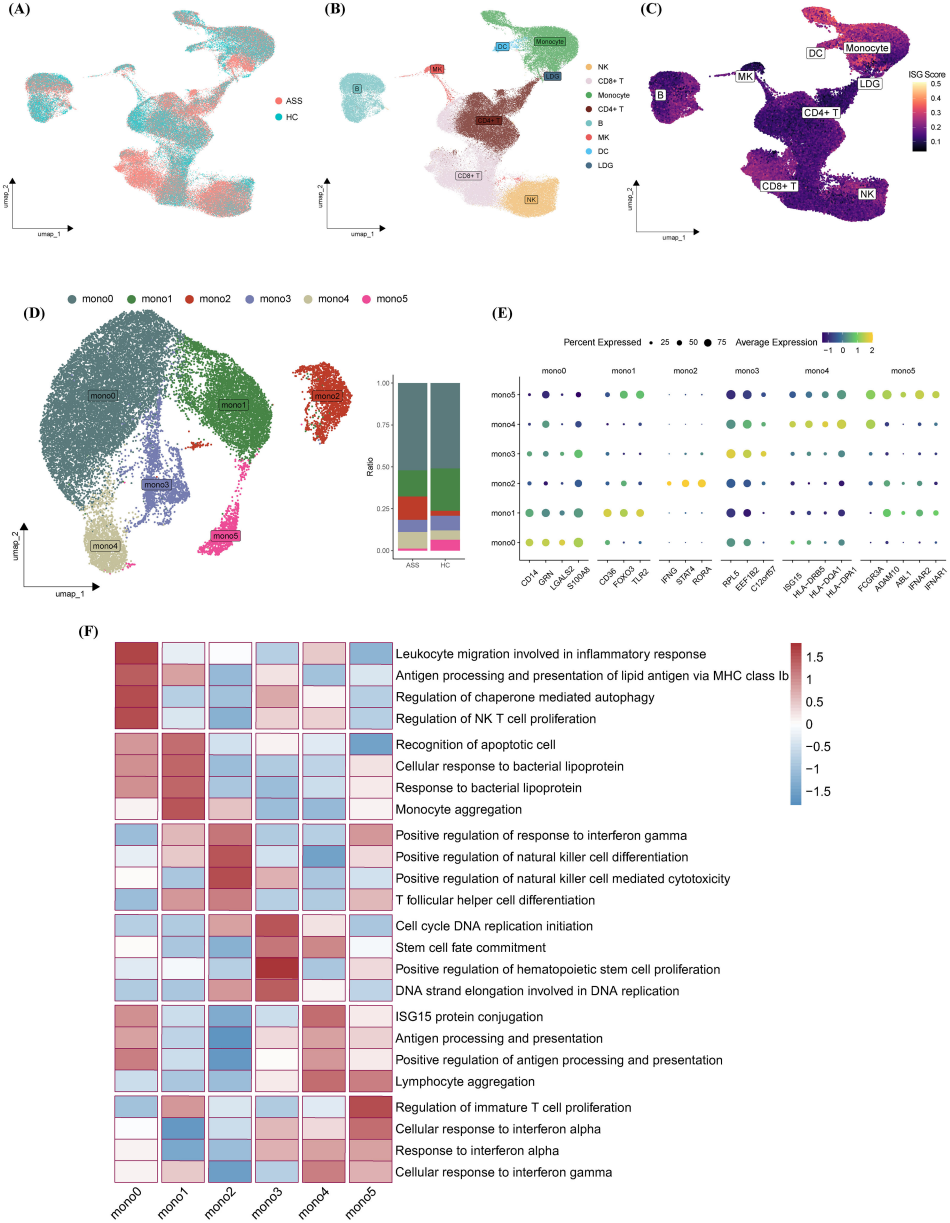
Functional and transcriptional profiling of monocyte subsets. **(A–C)** UMAP plots colored by **(A)** disease status, **(B)** cell type, and **(C)** ISG scores following integration of internal and external datasets. **(D)** Identification of six monocyte subsets (Mono0–Mono5) visualized on UMAP; bar plots indicate subset proportions across experimental groups. **(E)** Heatmap of marker gene expression across monocyte subsets. **(F)** GOBP-based heatmap highlighting representative biological processes enriched in each subset.

To enhance statistical power, we integrated our dataset with an external cohort (GSE190510), yielding a total of 126,026 cells for analysis. Consistent with our discovery findings, monocytes displayed the highest ISG activity across all immune lineages in the integrated dataset (**Figures 2A–C**). Subsequent subclustering identified six transcriptionally distinct monocyte subsets (Mono0–Mono5) (**Figure 2D**).

Classical monocytes (Mono0/1) expressed *CD14* along with genes related to leukocyte migration (e.g., *GRN*, *LGALS2*) and apoptotic recognition (e.g., *CD36*, *TLR2*). Mono2 was characterized by upregulation of *IFNG*, *STAT4*, and *RORA*, suggesting a proinflammatory trajectory involving Th1/Th17 polarization and NK cell activation. Mono3 showed proliferative signatures via ribosomal and translational machinery (e.g., *RPL5*, *EEF1B2*). Nonclassical *CD16^+^* monocytes (Mono4/5) displayed IFN-polarized features: Mono4 was enriched in antigen presentation (e.g., *ISG15*, *HLA*), while Mono5 showed IFN-adaptive potential through *IFNAR1/2* overexpression. Functional heatmap analysis revealed distinct gene expression and pathway enrichment across subsets, suggesting specialized roles in ASS-ILD pathogenesis (**Figures 2E, F**).

### 3.3 Monocyte pseudotime analysis reveals skewed inflammatory differentiation in ASS-ILD

**Figure 3:**
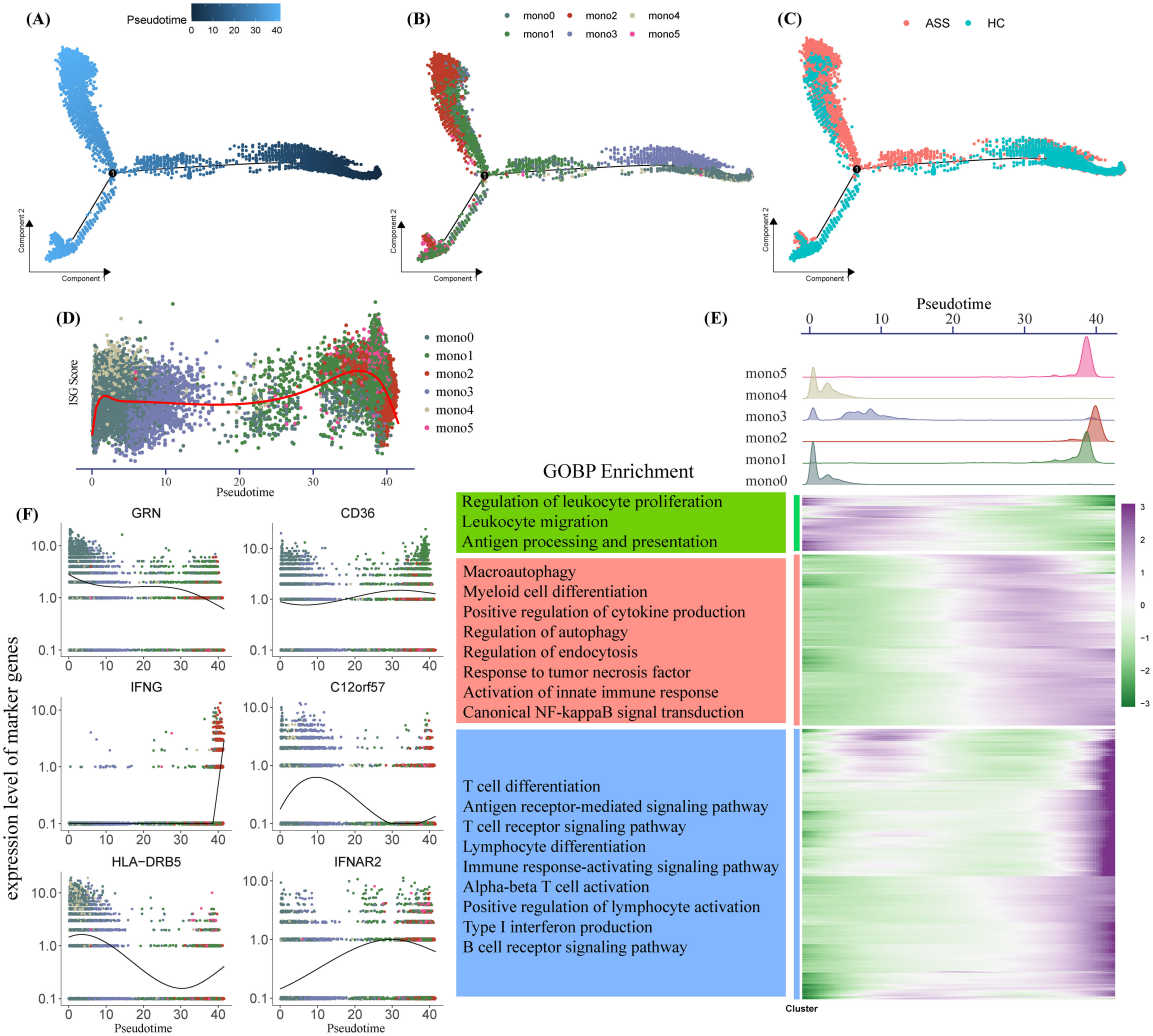
Pseudotime analysis of monocyte differentiation trajectories. **(A–C)** Monocle-inferred pseudotime trajectory of monocytes, colored by **(A)** pseudotime, **(B)** subset identity, and **(C)** disease state. Each dot represents a single cell. **(D, F)** Gene expression dynamics over pseudotime, showing ISG scores **(D)** and subtype-specific marker genes **(F)**, colored by monocyte subset. **(E)** Composite analysis: Top, density plots of monocyte subsets along pseudotime; bottom left, GOBP enrichment of pseudotime-regulated gene clusters; bottom right, heatmap of differentially expressed genes over pseudotime.

To delineate the developmental trajectory of monocyte subsets, we applied pseudotime inference using the Monocle algorithm. The trajectory branched from Mono0 toward two terminal fates—Mono1 and Mono2. Cells from ASS-ILD patients showed a preferential trajectory toward Mono2, while those from HCs were more diffusely distributed toward Mono0 and Mono1 (**Figure 3A, C**). Notably, ISG scores increased along the pseudotime axis (**Figure 3D**), supporting progressive interferon polarization.

Monocyte subset density across pseudotime revealed early-stage enrichment of Mono0, Mono3, and Mono4, with later stages dominated by Mono1, Mono2, and Mono5 (**Figure 3E**). Differential gene expression over pseudotime (adjusted p < 0.0001, logFC > 1) clustered into three groups enriched for leukocyte proliferation/migration, innate immunity, and adaptive immunity, respectively (**Figure 3F**). These results suggest that *IFNG^+^* monocytes (Mono2) accumulate at later pseudotime stages and may contribute to the transition from basal to inflammatory immune phenotypes in ASS-ILD.

### 3.4 IFN-γ and TNF signaling mediate monocyte-centric immune communication networks

**Figure 4:**
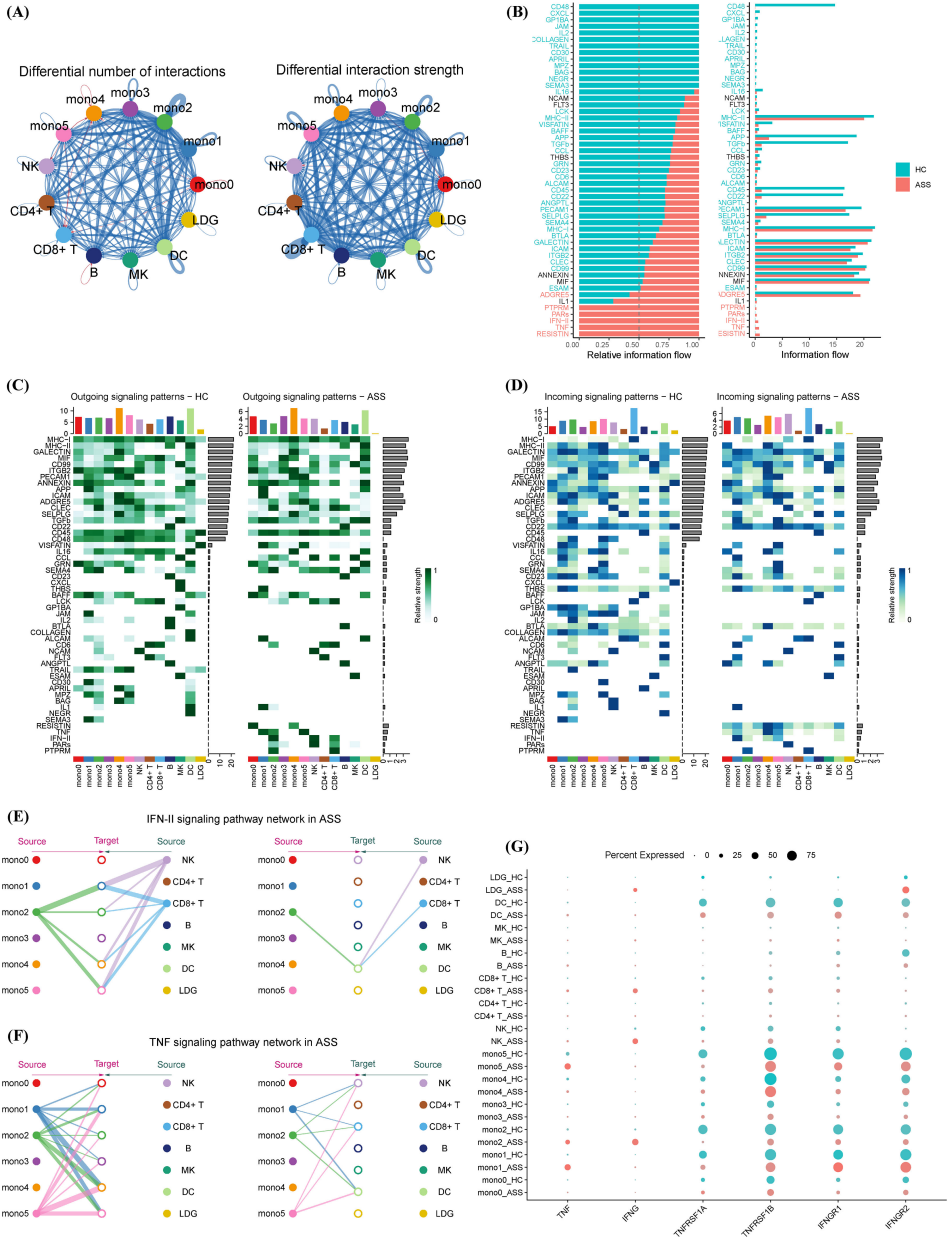
Cell–cell communication networks involving monocyte subsets in ASS-ILD and healthy controls. **(A)** Global interaction network comparing cell–cell communication strength between groups; red indicates ASS-ILD, blue indicates HCs; edge width corresponds to interaction strength. **(B)** Stacked bar plot displaying the distribution of specific signaling pathways under different conditions. **(C, D)** Heatmaps of secreted **(C)** and received **(D)** signaling interactions across immune cell types in ASS-ILD versus HCs. **(E, F)** Network diagrams showing IFN-γ (type II interferon) **(E)** and TNF **(F)** signaling pathways activated in ASS-ILD. **(G)** Dot plot comparing ligand and receptor expression levels for IFN-γ and TNF signaling between ASS-ILD and HC groups.

To explore intercellular signaling dynamics, we performed CellChat analysis to compare ligand–receptor interactions between ASS-ILD patients and HCs. Although global communication strength was reduced in ASS-ILD (**Figure 4A**), there was selective upregulation of type II interferon (IFN-γ) and tumor necrosis factor (TNF) signaling pathways (**Figure 4B**). Directional analysis revealed that Mono2, NK cells, and CD8^+^ T cells were dominant sources of IFN-γ and TNF signals, targeting monocyte subsets (Mono1/2/4/5) (**Figures 4C, D**).

Network visualization showed that IFN-γ signaling from Mono2, NK, and CD8^+^ T cells converged on downstream monocyte subsets, while TNF signals from Mono1, Mono2, and Mono5 regulated NK cells, CD8^+^ T cells, DCs, and other monocytes (**Figures 4E, F**). Ligand–receptor profiling indicated comparable receptor levels between groups but markedly elevated TNF-α and IFN-γ ligand expression in ASS-ILD (**Figure 4G**). These data reveal that IFN-γ and TNF signaling networks, driven in part by the Mono2 subset, amplify monocyte–immune crosstalk and may contribute to sustained inflammatory circuits in ASS-ILD.

### 3.5 Pathogenic transcription factor programs define monocyte subset polarization

**Figure 5:**
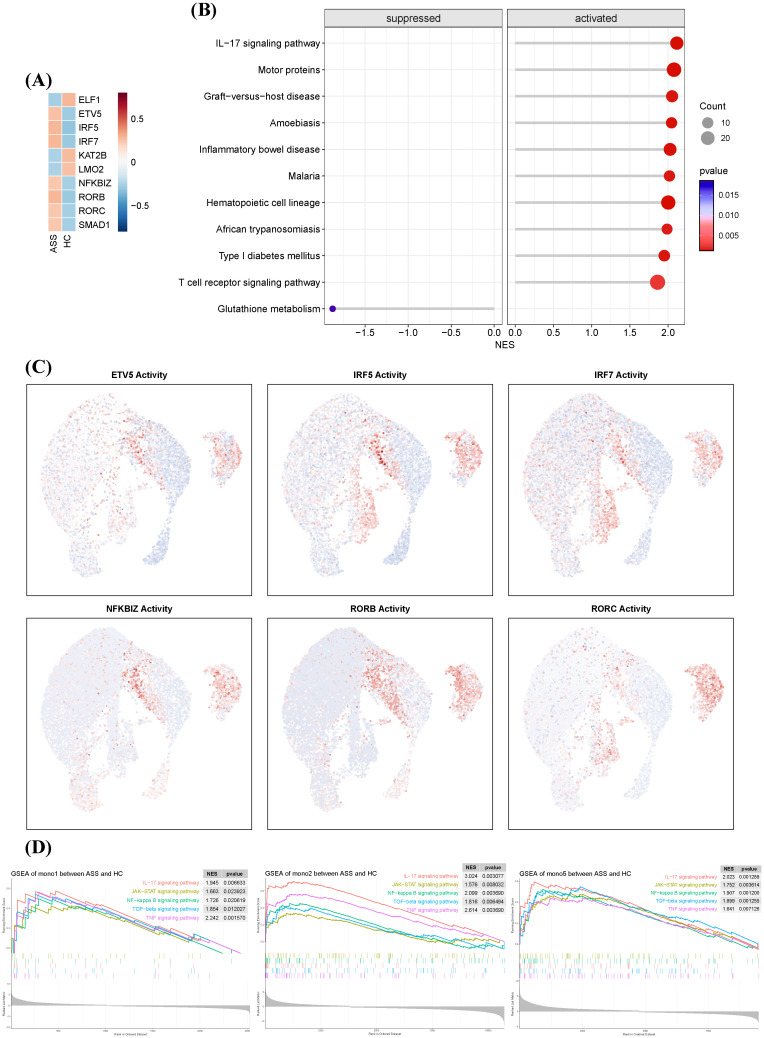
Transcriptional regulatory landscape of monocyte subsets in ASS-ILD. **(A)** Heatmap showing the top 10 most active transcription factors (TFs) in monocytes from ASS-ILD and HC samples, ranked by inferred activity. **(B)** Dot plot of the top 10 pathways enriched or depleted in ASS-ILD monocytes, identified by gene set enrichment analysis (GSEA). **(C)** UMAP plots displaying representative TF activity in monocyte subsets Mono1, Mono2, and Mono5. **(D)** GSEA enrichment plots showing significant upregulation of innate immune pathways in Mono1, Mono2, and Mono5 in ASS-ILD.

To identify regulatory drivers of monocyte subset behavior, we employed decoupleR-based inference using CollecTRI TF-target interaction data. Six transcription factors—*ETV5, IRF5, IRF7, RORB, RORC*, and *SMAD1*—showed the highest inferred activity in ASS-ILD monocytes (**Figure 5A**). These TFs are linked to immune polarization, interferon signaling, and fibrosis: ETV5 promotes Th1/Th17 responses via STAT3/STAT4 activation and IFN-γ/IL-17A production (19); IRF5 and IRF7 are canonical regulators of IFN pathways and macrophage activation (20, 21); RORB and RORC regulate autophagy and Th17 cell differentiation (22–24); SMAD1 mediates TGF-β signaling and contributes to fibrotic remodeling (25).

Gene set enrichment analysis (GSEA) of these TF targets showed enrichment in IL-17, T cell receptor, and inflammatory bowel disease pathways (**Figure 5B**). Notably, Mono2 exhibited the highest TF activity among monocyte subsets (**Figure 5C**). Pathway activity profiling revealed that Mono1, Mono2, and Mono5 in ASS-ILD were enriched in innate and adaptive immunity-related pathways, including IL-17, JAK-STAT, NF-κB, TGF-β, and TNF signaling (**Figure 5D**). These results position Mono2 as a transcriptionally primed subset with proinflammatory and profibrotic potential.

### 3.6 Validation of key genes by qRT-PCR and an external dataset

**Figure 6:**
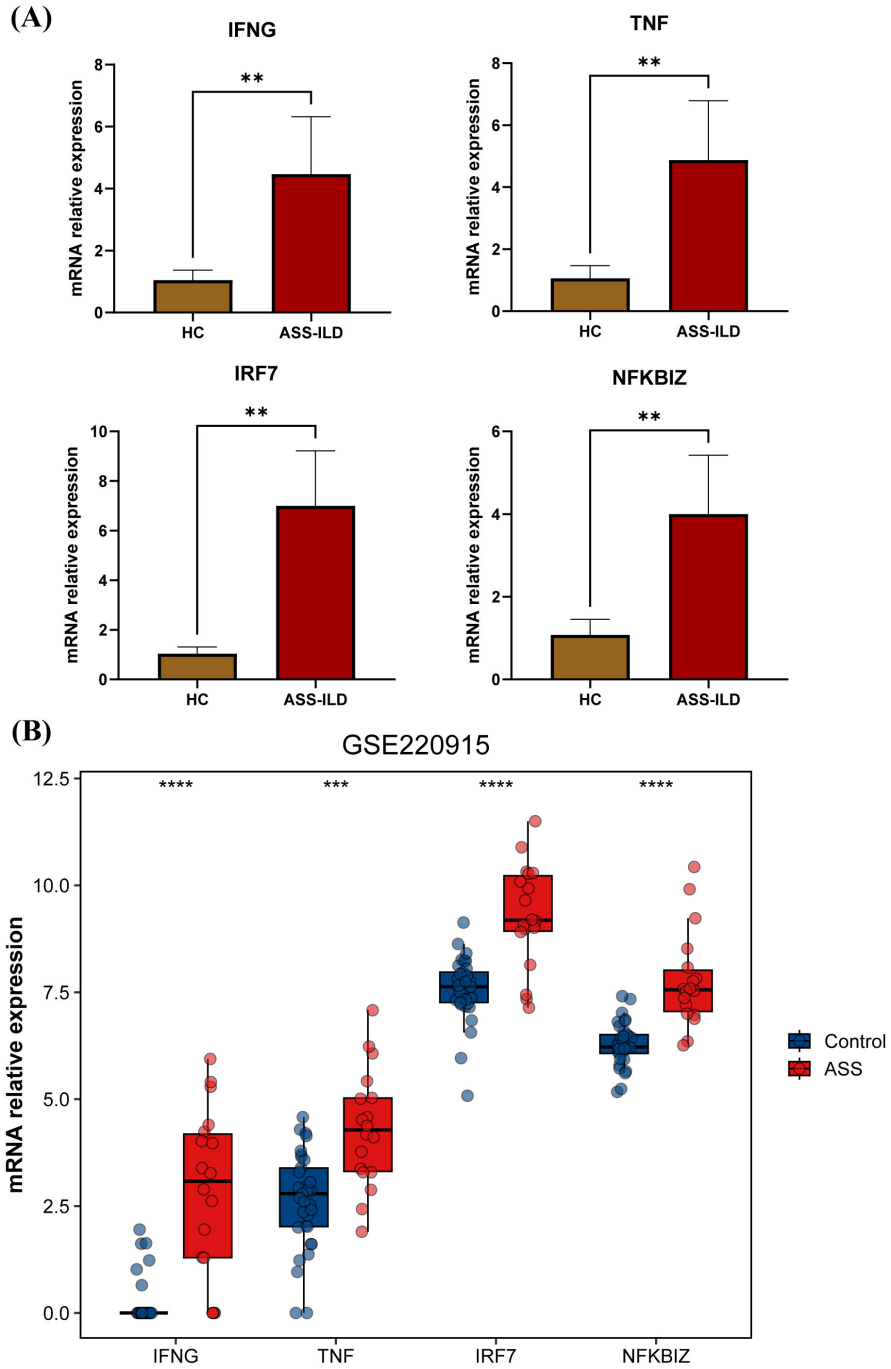
Validation of key genes by qRT-PCR and an external dataset. **(A)** Bar plot showing the relative expression levels of *IFNG*, *TNF*, *IRF7*, and *NFKBIZ* in PBMCs from ASS-ILD patients and healthy controls (HC) based on qRT-PCR results. **(B)** Bar plot showing the relative expression levels of *IFNG*, *TNF*, *IRF7*, and *NFKBIZ* in muscle tissue from ASS-ILD patients and healthy controls (HC) based on the external dataset GSE220915.

To further validate our findings, we assessed the expression of key genes, including *IFNG*, *TNF*, *IRF7*, and *NFKBIZ*. The qRT-PCR results revealed that the expression of these genes was significantly higher in the PBMCs of ASS-ILD patients compared to healthy controls (**Figure 6A**). Additionally, we incorporated a bulk RNA-seq dataset from muscle biopsy samples, which included 13 ASS patients and 33 healthy controls. Similar to the findings in PBMCs, the expression levels of these four genes were elevated in the muscle tissue of patients, compared to healthy controls (**Figure 6B**). This suggests that these genes are highly expressed not only in peripheral blood but also in affected tissues, further confirming the reliability of our results.

## 4 Discussion

The interferon (IFN) family, comprising critical effector molecules of the innate immune system, plays a central role in host defense and immune homeostasis (26). Aberrant activation of the IFN signaling pathway is a well-established hallmark of classical autoimmune diseases such as systemic lupus erythematosus (SLE) and Sjögren’s syndrome (SS) (27), and is increasingly recognized as a key driver in the pathogenesis of idiopathic inflammatory myopathy-associated interstitial lung disease (IIM-ILD) (28). In the present study, we employed single-cell RNA sequencing (scRNA-seq) to comprehensively characterize peripheral blood mononuclear cells (PBMCs) in patients with antisynthetase syndrome-associated interstitial lung disease (ASS-ILD), with a focus on IFN-related cellular and molecular alterations.

A cross-cohort analysis of scRNA-seq data revealed that monocytes exhibited the highest IFN-stimulated gene (ISG) scores among all immune cell populations. Monocytes, essential players in innate immunity (29), are frequently dysregulated in autoimmune diseases (24), a finding supported by our functional enrichment analyses of monocyte-specific transcriptomes. Traditionally, monocytes are categorized into classical (CD14^+^ CD16^-^), intermediate (CD14^+^ CD16^+^), and nonclassical (CD14^-^ CD16^+^) subsets based on CD14 and CD16 expression (30). However, our data further subdivided these into six transcriptionally distinct clusters: mono0 and mono1 (classical), mono2 and mono3 (intermediate), and mono4 and mono5 (nonclassical). Notably, mono2, enriched in ASS-ILD patients, exhibited elevated *IFNG* expression (**Figure 2D**). While IFN-γ is classically produced by NK and T cells, accumulating evidence suggests that monocytes, macrophages, and B cells are also capable of IFN-γ production (31, 32). Our findings thus highlight monocytes, particularly the mono2 subset, as potential contributors to IFN-γ production in ASS-ILD.

In parallel, mono5 showed high expression of *IFNAR1* and *IFNAR2*, suggesting increased sensitivity to type I IFN signaling and a potential role in bridging innate and adaptive immune responses (**Figures 2E, F**). Trajectory analysis using Monocle further indicated that accumulation of *IFNG^+^* monocytes (mono2) may drive proinflammatory microenvironment remodeling in ASS-ILD.

Cell–cell communication analysis revealed significant alterations in TNF and IFN-γ (type II IFN) signaling between ASS-ILD patients and healthy controls. Monocytes, NK cells, and T cells were identified as the principal mediators of these signals, consistent with previous reports (33).Specifically, monocyte subsets mono1, mono2, and mono5 were the primary transmitters of TNF signals, interacting with NK cells, T cells, dendritic cells (DCs), and other monocytes. For IFN-γ signaling, mono2 emerged as the dominant transmitter, targeting DCs and monocytes. Importantly, monocytes functioned both as senders and receivers in these signaling cascades.

The biological relevance of these findings is underscored by the central roles of TNF-α and IFN-γ in autoimmune inflammation and immune regulation. TNF-α promotes apoptosis, enhances proinflammatory cytokine release, and contributes to epithelial and vascular injury (34). It also induces M1 macrophage polarization and IL-6 production, exacerbating inflammatory responses (35). The dual participation of monocytes in both TNF and IFN-γ signaling suggests a critical role in amplifying cytokine cascades and driving ILD progression.

To explore the transcriptional regulation underlying these effects, we identified key transcription factors (TFs) with elevated activity in ASS-ILD monocytes, including *ETV5, IRF5, IRF7, RORB, RORC*, and *SMAD1*. Notably, ETV5, IRF5, and IRF7 are closely associated with interferon signaling (19–21). In addition, ETV5 and RORC have been linked to IL-17A production, a proinflammatory cytokine that bridges innate and adaptive immunity and is implicated in systemic autoimmunity (19, 23, 36). SMAD1, activated by TGF-β signaling, promotes proinflammatory and fibrotic responses by recruiting immune cells and fibroblasts to the lung parenchyma (37). Consistent with these roles, GSEA revealed enrichment of IL-17, JAK-STAT, NF-κB, TGF-β, and TNF pathways in mono1, mono2, and mono5 (**Figure 5D**), suggesting a regulatory network that sustains pulmonary inflammation and fibrosis—key pathological features of ILD.

Taken together, our data identify monocytes as central orchestrators of immune dysregulation in ASS-ILD, integrating cytokine signaling and transcriptional reprogramming to drive disease progression. These findings offer a cellular and molecular framework for understanding monocyte-driven inflammation and highlight potential targets for therapeutic intervention.

This study has several limitations. First, although we incorporated an external dataset to enhance the robustness of cross-cohort analysis, the overall sample size remains modest, limiting statistical power and generalizability. Future studies should aim to validate these findings in larger, independent cohorts. Second, while we identified an *IFNG^+^* monocyte subset (mono2) characterized by *CD14^low^CD16^low^* expression, functional validation and mechanistic characterization of this population are warranted. Third, our analysis was based solely on PBMCs, without access to lung tissue or bronchoalveolar lavage specimens. This precludes direct insight into tissue-resident immune responses and local inflammation. Further studies incorporating *in vitro* and *in vivo* models, as well as tissue-specific samples, are essential to fully elucidate the functional roles and signaling programs of monocyte subsets in ASS-ILD pathogenesis. Although this study is based solely on scRNA-seq profiling, the convergence of multiple analytical pipelines including AUCell, Monocle, and CellChat enhances the robustness of the inferred mechanisms. These findings provide a rationale for further experimental validation and the development of targeted therapies.

## 5 Conclusion

In summary, our study identifies monocytes as the key immune cell type associated with heightened interferon (IFN) signaling in the peripheral blood of patients with antisynthetase syndrome-associated interstitial lung disease (ASS-ILD). These monocytes, particularly specific transcriptionally defined subsets, engage in aberrant IFN-γ (type II IFN) and TNF signaling, which appear to play central roles in the pathogenesis of ASS-ILD. Beyond delineating pathogenic mechanisms, our findings reveal potential therapeutic targets and pathways—including IFN-related transcription factors and cytokine signaling networks—that may inform the development of precision therapies. These insights offer a conceptual and molecular framework for future clinical studies aimed at improving treatment strategies for ASS-ILD.

## Data Availability

The datasets presented in this study can be found in online repositories. The names of the repository/repositories and accession number(s) can be found below: https://ngdc.cncb.ac.cn/gsa-human/, HRA011247, HRA010146, and HRA000916.

## Supplementary material

Supplementary Figure 1 Representative high-resolution computed tomography (HRCT) images of three patients with ASS-ILD, showing typical radiologic features.

Supplementary Table 1 Clinical manifestations and laboratory characteristics of the three ASS-ILD patients in the discovery cohort.

Supplementary Table 2 List of interferon-stimulated genes (ISGs) curated from the Interferome database used for AUCell scoring.

## References

[1] Zhan X Yan W Wang Y Li Q Shi X Gao Y. Clinical features of anti-synthetase syndrome associated interstitial lung disease: a retrospective cohort in China. BMC Pulm Med. (2021) 21:57. doi: 10.1186/s12890-021-01399-5, PMID: 33579248 PMC7881640

[2] Hervier B Uzunhan Y. Inflammatory myopathy-related interstitial lung disease: from pathophysiology to treatment. Front Med (Lausanne). (2019) 6:326. doi: 10.3389/fmed.2019.00326, PMID: 32010700 PMC6978912

[3] Wong AW Ryerson CJ Guler SA. Progression of fibrosing interstitial lung disease. Respir Res. (2020) 21:32. doi: 10.1186/s12931-020-1296-3, PMID: 31996266 PMC6988233

[4] Hervier B Benveniste O. Clinical heterogeneity and outcomes of antisynthetase syndrome. Curr Rheumatol Rep. (2013) 15:349. doi: 10.1007/s11926-013-0349-8, PMID: 23794106

[5] Gasparotto M Franco C Zanatta E Ghirardello A Zen M Iaccarino L. The interferon in idiopathic inflammatory myopathies: Different signatures and new therapeutic perspectives. A literature review. Autoimmun Rev. (2023) 22:103334. doi: 10.1016/j.autrev.2023.103334, PMID: 37068699

[6] McNab F Mayer-Barber K Sher A Wack A O’Garra A. Type I interferons in infectious disease. Nat Rev Immunol. (2015) 15:87–103. doi: 10.1038/nri3787, PMID: 25614319 PMC7162685

[7] Gallay L Mouchiroud G Chazaud B. Interferon-signature in idiopathic inflammatory myopathies. Curr Opin Rheumatol. (2019) 31:634–42. doi: 10.1097/BOR.0000000000000653, PMID: 31464706

[8] Solomon J Swigris JJ Brown KK. Myositis-related interstitial lung disease and antisynthetase syndrome. Jornal brasileiro pneumologia. (2011) 37:100–9. doi: 10.1590/S1806-37132011000100015, PMID: 21390438 PMC3676869

[9] Zhu L Cao Z Wang S Zhang C Fang L Ren Y. Single-cell transcriptomics reveals peripheral immune responses in anti-synthetase syndrome-associated interstitial lung disease. Front Immunol. (2022) 13:804034. doi: 10.3389/fimmu.2022.804034, PMID: 35250976 PMC8891123

[10] Ashburner M Ball CA Blake JA Botstein D Butler H Cherry JM. Gene ontology: tool for the unification of biology. The Gene Ontology Consortium. Nat Genet. (2000) 25:25–9. doi: 10.1038/75556, PMID: 10802651 PMC3037419

[11] Kanehisa M Goto S. KEGG: kyoto encyclopedia of genes and genomes. Nucleic Acids Res. (2000) 28:27–30. doi: 10.1093/nar/28.1.27, PMID: 10592173 PMC102409

[12] Yu G Wang LG Han Y He QY. clusterProfiler: an R package for comparing biological themes among gene clusters. Omics: J Integr Biol. (2012) 16:284–7. doi: 10.1089/omi.2011.0118, PMID: 22455463 PMC3339379

[13] Aibar S González-Blas CB Moerman T Huynh-Thu VA Imrichova H Hulselmans G. SCENIC: single-cell regulatory network inference and clustering. Nat Methods. (2017) 14:1083–6. doi: 10.1038/nmeth.4463, PMID: 28991892 PMC5937676

[14] Hänzelmann S Castelo R Guinney J. GSVA: gene set variation analysis for microarray and RNA-seq data. BMC Bioinf. (2013) 14:7. doi: 10.1186/1471-2105-14-7, PMID: 23323831 PMC3618321

[15] Jin S Guerrero-Juarez CF Zhang L Chang I Ramos R Kuan CH. Inference and analysis of cell-cell communication using CellChat. Nat Commun. (2021) 12:1088. doi: 10.1038/s41467-021-21246-9, PMID: 33597522 PMC7889871

[16] Badia IMP Vélez Santiago J Braunger J Geiss C Dimitrov D Müller-Dott S. decoupleR: ensemble of computational methods to infer biological activities from omics data. Bioinform Adv. (2022) 2:vbac016. doi: 10.1093/bioadv/vbac016, PMID: 36699385 PMC9710656

[17] Müller-Dott S Tsirvouli E Vazquez M Ramirez Flores RO Badia IMP Fallegger R. Expanding the coverage of regulons from high-confidence prior knowledge for accurate estimation of transcription factor activities. Nucleic Acids Res. (2023) 51:10934–49. doi: 10.1093/nar/gkad841, PMID: 37843125 PMC10639077

[18] Casal-Dominguez M Pinal-Fernandez I Pak K Muñoz-Braceras S Milisenda JC Torres-Ruiz J. Coordinated local RNA overexpression of complement induced by interferon gamma in myositis. Sci Rep. (2023) 13:2038. doi: 10.1038/s41598-023-28838-z, PMID: 36739295 PMC9899209

[19] Shi Y Dai S Qiu C Wang T Zhou Y Xue C. MicroRNA-219a-5p suppresses intestinal inflammation through inhibiting Th1/Th17-mediated immune responses in inflammatory bowel disease. Mucosal Immunol. (2020) 13:303–12. doi: 10.1038/s41385-019-0216-7, PMID: 31628427

[20] Sgarbanti M Marsili G Remoli AL Orsatti R Battistini A. IRF-7: new role in the regulation of genes involved in adaptive immunity. Ann N Y Acad Sci. (2007) 1095:325–33. doi: 10.1196/annals.1397.036, PMID: 17404045

[21] Barnes BJ Moore PA Pitha PM. Virus-specific activation of a novel interferon regulatory factor, IRF-5, results in the induction of distinct interferon alpha genes. J Biol Chem. (2001) 276:23382–90. doi: 10.1074/jbc.M101216200, PMID: 11303025

[22] Yan G Lei H He M Gong R Wang Y He X. Melatonin triggers autophagic cell death by regulating RORC in Hodgkin lymphoma. BioMed Pharmacother. (2020) 123:109811. doi: 10.1016/j.biopha.2020.109811, PMID: 31924597

[23] Castro G Liu X Ngo K De Leon-Tabaldo A Zhao S Luna-Roman R. RORγt and RORα signature genes in human Th17 cells. PloS One. (2017) 12:e0181868. doi: 10.1371/journal.pone.0181868, PMID: 28763457 PMC5538713

[24] Ferreté-Bonastre AG Martínez-Gallo M Morante-Palacios O Calvillo CL Calafell-Segura J Rodríguez-Ubreva J. Disease activity drives divergent epigenetic and transcriptomic reprogramming of monocyte subpopulations in systemic lupus erythematosus. Ann Rheum Dis. (2024) 83:865–78. doi: 10.1136/ard-2023-225433, PMID: 38413168

[25] Guan R Yuan L Li J Wang J Li Z Cai Z. Bone morphogenetic protein 4 inhibits pulmonary fibrosis by modulating cellular senescence and mitophagy in lung fibroblasts. Eur Respir J. (2022) 60(6):2102307. doi: 10.1183/13993003.02307-2021, PMID: 35777761 PMC9808813

[26] Crow MK Olferiev M Kirou KA. Targeting of type I interferon in systemic autoimmune diseases. Transl Res. (2015) 165:296–305. doi: 10.1016/j.trsl.2014.10.005, PMID: 25468480 PMC4306610

[27] Drougkas K Skarlis C Mavragani C. Type I interferons in systemic autoimmune rheumatic diseases: pathogenesis, clinical features and treatment options. Mediterr J Rheumatol. (2024) 35:365–80. doi: 10.31138/mjr.270324.tis, PMID: 39193187 PMC11345602

[28] Bolko L Jiang W Tawara N Landon-Cardinal O Anquetil C Benveniste O. The role of interferons type I, II and III in myositis: A review. Brain Pathol. (2021) 31:e12955. doi: 10.1111/bpa.12955, PMID: 34043262 PMC8412069

[29] Ma WT Gao F Gu K Chen DK. The role of monocytes and macrophages in autoimmune diseases: A comprehensive review. Front Immunol. (2019) 10:1140. doi: 10.3389/fimmu.2019.01140, PMID: 31178867 PMC6543461

[30] Ożańska A Szymczak D Rybka J. Pattern of human monocyte subpopulations in health and disease. Scand J Immunol. (2020) 92:e12883. doi: 10.1111/sji.12883, PMID: 32243617

[31] Mezouar S Mege JL. Changing the paradigm of IFN-γ at the interface between innate and adaptive immunity: Macrophage-derived IFN-γ. J Leukoc Biol. (2020) 108:419–26. doi: 10.1002/JLB.4MIR0420-619RR, PMID: 32531848

[32] Fillatreau S. Pathogenic functions of B cells in autoimmune diseases: IFN-γ production joins the criminal gang. Eur J Immunol. (2015) 45:966–70. doi: 10.1002/eji.201545544, PMID: 25727209

[33] Ding J Li Y Wang Z Han F Chen M Du J. A distinct immune landscape in anti-synthetase syndrome profiled by a single-cell genomic study. Front Immunol. (2024) 15:1436114. doi: 10.3389/fimmu.2024.1436114, PMID: 39512337 PMC11540782

[34] Abianeh HS Kesharwani P Sahebkar A. The use of aptamers as therapeutic inhibitors and biosensors of TNF-alpha. Int J Biol Macromol. (2025) 306:141202. doi: 10.1016/j.ijbiomac.2025.141202, PMID: 39971069

[35] Ivashkiv LB. IFNγ: signalling, epigenetics and roles in immunity, metabolism, disease and cancer immunotherapy. Nat Rev Immunol. (2018) 18:545–58. doi: 10.1038/s41577-018-0029-z, PMID: 29921905 PMC6340644

[36] Zhang J Shen M. The role of IL-17 in systemic autoinflammatory diseases: mechanisms and therapeutic perspectives. Clin Rev Allergy Immunol. (2025) 68:27. doi: 10.1007/s12016-025-09042-5, PMID: 40074883

[37] Huo R Huang X Yang Y Yang Y Lin J. Potential of resveratrol in the treatment of interstitial lung disease. Front Pharmacol. (2023) 14:1139460. doi: 10.3389/fphar.2023.1139460, PMID: 37089962 PMC10117935

[38] Jiang S Li H Zhang L Mu W Zhang Y Chen T. Generic Diagramming Platform (GDP): a comprehensive database of high-quality biomedical graphics. Nucleic Acids Res. (2025) 53:D1670–d6. doi: 10.1093/nar/gkae973, PMID: 39470721 PMC11701665

