# Supplementary figures and images for: Monocyte-driven IFN and TNF programs orchestrate inflammatory networks in antisynthetase syndrome-associated interstitial lung disease
Source: Front Immunol. 2025 Oct 23;16:1652999. doi: 10.3389/fimmu.2025.1652999 (PMC12589074; doi:10.3389/fimmu.2025.1652999)

ASS1

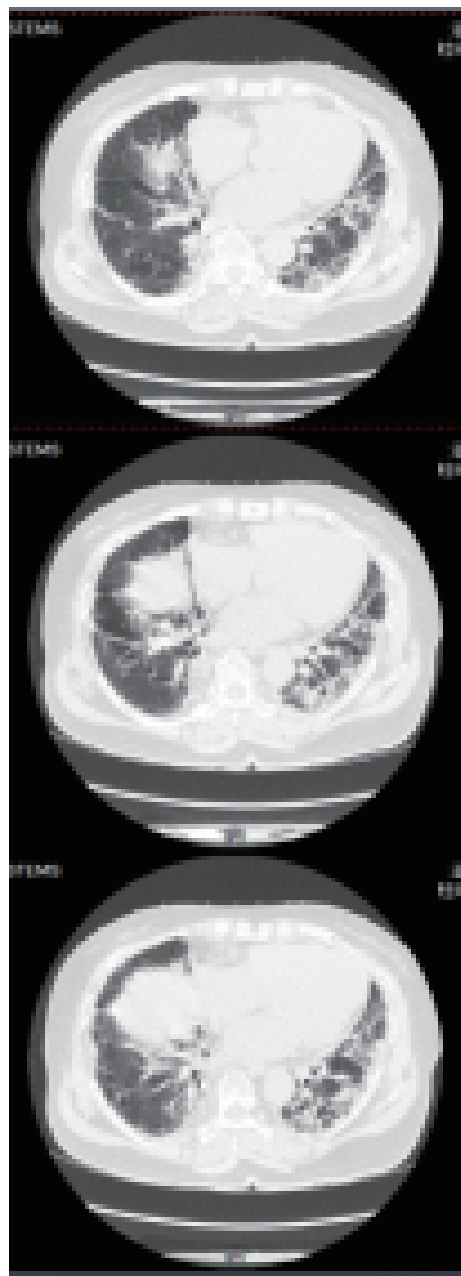

ASS2

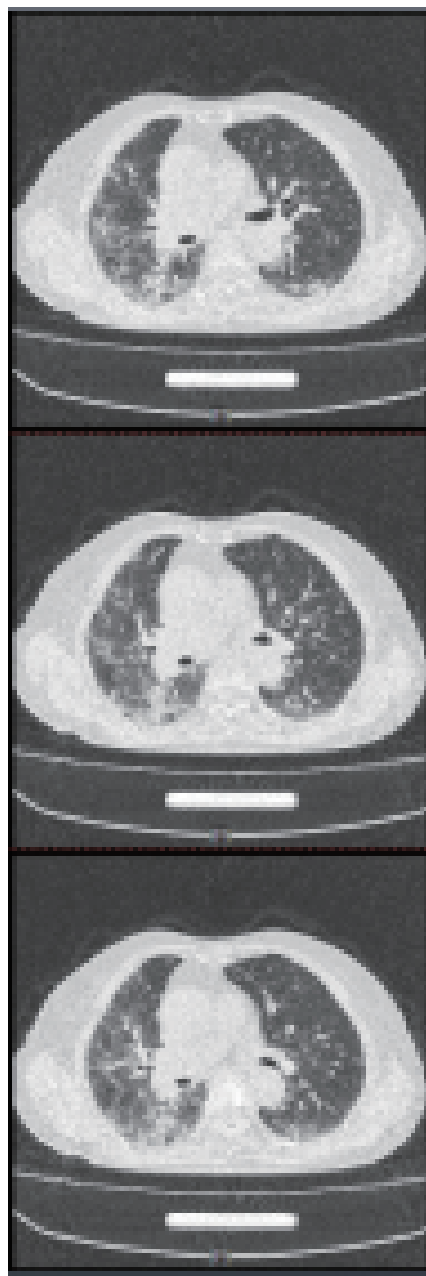

ASS3

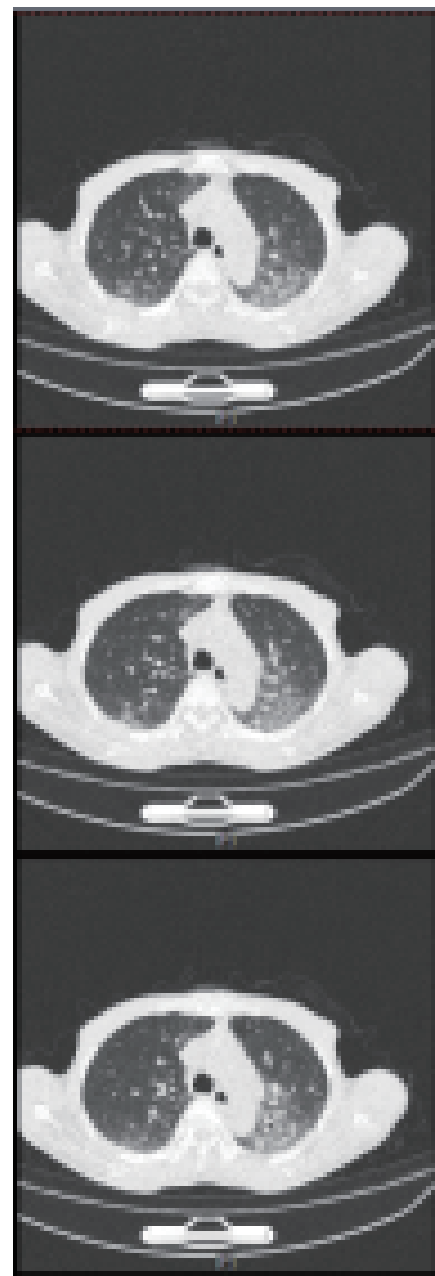

Supplement: Supplementary Figure 1 — Representative high-resolution computed tomography (HRCT) images of three patients with ASS-ILD, showing typical radiologic features. [file DataSheet1.pdf]
